# Supplementary material for: Identification of the Multiresistance Gene poxtA in Oxazolidinone-Susceptible Staphylococcus haemolyticus and Staphylococcus saprophyticus of Pig and Feed Origins
Source: Pathogens. 2021 May 14;10(5):601. doi: 10.3390/pathogens10050601 (PMC8156375; doi:10.3390/pathogens10050601)
Supplement: Supplementary file 1 [file pathogens-10-00601-s001.zip › Figure S1.pdf]

Supplementary data

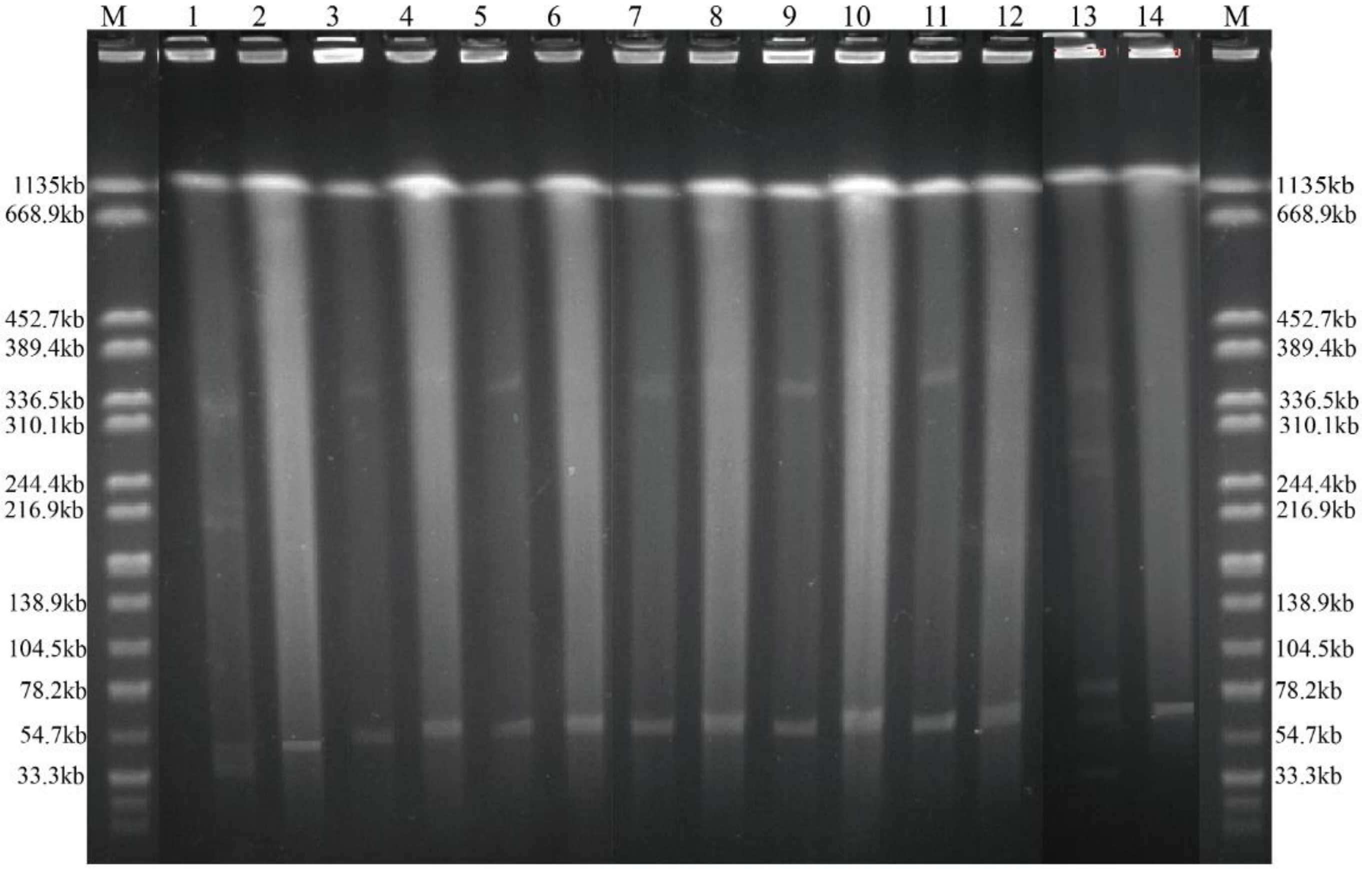

Figure S1. Location of the *poxtA*-carrying plasmids in seven CoNS isolates and corresponding electrotransformants by S1-PFGE. Lanes M contain the *Xba*I pattern of *Salmonella braenderup* H9812 with the fragment sizes given in kilobases on the left-hand and right-hand sides; lanes 1 to 14 represent GDH8C97P, RN4220/pH97, GDY8P33P, RN4220/pY33, GDY8P50P, RN4220/pY50, GDY8P58P, RN4220/pY58, GDY8P60P, RN4220/pY60, GDY8P80P, RN4220/pY80, GDY8P136P and RN4220/pY136, respectively.
